# Adaptive genetic markers discriminate migratory runs of Chinook salmon (*Oncorhynchus tshawytscha*) amid continued gene flow

**DOI:** 10.1111/eva.12095

**Published:** 2013-09-10

**Authors:** Kathleen G O'Malley, Dave P Jacobson, Ryon Kurth, Allen J Dill, Michael A Banks

**Affiliations:** 1 Department of Fisheries and Wildlife, Coastal Oregon Marine Experiment Station, Hatfield Marine Science Center, Oregon State UniversityNewport, OR, USA; 2 California Department of Water Resources, Division of Environmental ServicesOroville, CA, USA; 3 California Department of Fish and Game, Feather River HatcheryOroville, CA, USA

**Keywords:** captive populations, conservation biology, conservation genetics, ecological genetics, fisheries management, hybridization, life history evolution, population genetics

## Abstract

Neutral genetic markers are routinely used to define distinct units within species that warrant discrete management. Human-induced changes to gene flow however may reduce the power of such an approach. We tested the efficiency of adaptive versus neutral genetic markers in differentiating temporally divergent migratory runs of Chinook salmon (*Oncorhynchus tshawytscha*) amid high gene flow owing to artificial propagation and habitat alteration. We compared seven putative migration timing genes to ten microsatellite loci in delineating three migratory groups of Chinook in the Feather River, CA: offspring of fall-run hatchery broodstock that returned as adults to freshwater in fall (fall run), spring-run offspring that returned in spring (spring run), and fall-run offspring that returned in spring (FRS). We found evidence for significant differentiation between the fall and federally listed threatened spring groups based on divergence at three circadian clock genes (*OtsClock1b*, *OmyFbxw11,* and *Omy1009UW*), but not neutral markers. We thus demonstrate the importance of genetic marker choice in resolving complex life history types. These findings directly impact conservation management strategies and add to previous evidence from Pacific and Atlantic salmon indicating that circadian clock genes influence migration timing.

## Introduction

A major effort in conservation biology is directed toward defining units within species that are sufficiently differentiated to require discrete management (Frankham 2010). Identifying such conservation units (CUs) is an essential first step so that managers and policy makers know the boundaries of the populations that they are trying to conserve (Funk et al. 2012). Management strategies can then be developed to effectively target CUs to promote population growth, avoid exploitation, and develop reintroduction strategies (Allendorf et al. 2010).

The two most frequently discussed conservation units are evolutionary significant units (ESUs) and management units (MUs). An ESU can broadly be defined as a population or group of populations that warrant separate management because of high genetic and ecological distinctiveness (reviewed in Funk et al. 2012). Management units are typically defined as demographically independent populations whose population dynamics (e.g., population growth rate) depend largely on local birth and death rates rather than on immigration (Moritz 1994). Due to the low connectivity among populations, each unit should be monitored and managed separately (Taylor and Dizon 1999). As population structure is typically assessed by estimating divergence in the allele frequencies at neutral genetic markers (i.e., microsatellite loci), this class of genetic markers is routinely used to delineate both ESUs (Small et al. 1998) and MUs (Palsbøll et al. 2006).

However, human management of biological systems (e.g., artificial propagation and habitat alteration) has the potential to increase the rate of gene flow among CUs such that formerly diagnostic neutral markers provide limited power to discriminate populations. This may affect our ability to identify ESUs with distinct local adaptations that represent an important evolutionary legacy of a species (Waples 1991). Adaptive markers (e.g., *F*_st_ outliers or specific genes of known function) might, however, prove to be a better discriminatory tool when gene flow is high. Understanding adaptive differences among units is critical when prioritizing which populations to focus management decisions on if resources are limited or when deciding which populations to use for translocation and supplementation efforts (Funk et al. 2012).

In this study, we compare the performance of neutral versus adaptive markers to detect genetic differentiation among temporally divergent migratory runs of Chinook salmon (*Oncorhynchus tshawystcha*) that represent two different ESUs (NOAA 2005). Chinook salmon undertake an extensive oceanic feeding migration prior to returning to their natal freshwater environments to breed (Groot and Margolis 1991). Their high homing fidelity promotes reproductive isolation by distance, while the persistence of multiple seasonal migratory runs within single river systems may promote isolation by time. Selectively neutral markers have thus generally provided an effective means for delineating ESUs, a decisive classification in salmon conservation management (reviewed in Waples et al. 2004).

Artificial propagation and alteration of the physical landscape have resulted in human-induced changes to gene flow such that characteristically diagnostic neutral microsatellite markers may not discriminate seasonally migratory runs of Chinook salmon (Banks et al. 2000; Hedgecock et al. 2001; O'Malley et al. 2007). For example, in the Feather River, CA, spring-run fish enter freshwater in a reproductively immature state many months prior to spawning, which enabled them historically to reach high-elevation breeding grounds only accessible during peak springwater flows. In contrast, fall-run fish exploit the highly productive marine environment for growth and returned with well-developed gonads to spawn shortly after entering the lower stretches of the river. Construction of a hydropower dam in the early 1960s, however, eliminated this historical spring-run spawning habitat thereby eradicating the spatial component of reproductive isolation between spring- and fall-run migrants. To mitigate for this habitat loss, the Feather River Hatchery (FRH) was constructed in 1967. Hybridization of some phenotypically spring and fall-run Chinook continued to occur during hatchery production until the implementation of a tagging program in 2006. This program (detailed in the Material and methods) provided the means to isolate and collect only early-returning migrants for the spring-run broodstock and initiated the effort to preserve the phenotypic/genotypic characteristics of the spring run, a key component of the federally listed ‘threatened' Central Valley spring Chinook salmon ESU (NOAA 2005).

The timing of adult migration in salmonid fishes is known to be under strong genetic control (Hendry and Day 2005; Carlson and Seamons 2008). Recent studies suggest that circadian clock genes, which are primarily entrained by photoperiod, provide a molecular mechanism for long-term timekeeping to forecast the optimal timing of season-specific activities (Froy et al. 2003; Lincoln et al. 2003; Davie et al. 2009; Ikegami and Yoshimura 2012), As salmon migration timing is primarily an adaptation to long-term average conditions (Robards and Quinn 2002), photoperiod is believed to be a key long-term, stable environmental cue that fish use to coordinate their population-specific migratory runs with seasonally varying conditions (Quinn and Adams 1996). In a previous study, we found evidence for two genetically distinct migratory runs of Chinook salmon in the Feather River based on variation at two candidate markers for run timing; the circadian clock gene *OtsClock1b* and *Ots515NWFSC*, a microsatellite marker linked to a quantitative trait loci for spawning time and body weight in rainbow trout (*O. mykiss*) (O'Malley et al. 2007). Subsequent studies further suggest that *OtsClock1b* may mediate the timing of migration among other Pacific salmon species (O'Malley and Banks 2008b; O'Malley et al. 2010a,2010b).

The primary goal of this study was to test whether adaptive markers provide the power to discriminate between migratory runs of Chinook salmon amid continued gene flow. We evaluated six circadian clock gene markers *OtsClock1b*, *Cryptochrome2b.2*, *Cryptochrome2b.3*, *Cryptochrome3*, *OmyFbxw1*, *Omy1009UW,* and *Ots515NWFSC* as potential management tools to inform current hatchery practices and incorporate into a long-term monitoring plan, assessing the effectiveness of the tagging program. Realization of the FRH tagging program enabled us to identify three adult migratory groups that returned to the river in 2009: offspring of fall-run hatchery broodstock that returned as adults in the fall (fall run), offspring of spring-run hatchery broodstock that returned as adults in the spring (spring run), and offspring of fall-run hatchery broodstock that returned as adults in the spring (fall return spring, FRS). First, we used ten presumably neutral microsatellite loci to test for genetic divergence due to drift among the three migratory groups. We predicted that amid high gene flow, the fall, spring, and FRS groups would be genetically indistinct. We then used the seven adaptive markers to test for genetic differentiation among the three migratory groups. We predicted that the fall versus spring and fall versus FRS groups would be genetically distinct, whereas the spring and FRS fish would be indistinguishable (i.e., positive control).

## Materials and methods

### Study system and samples

Historically, the Feather River, California, supported both fall and spring Chinook salmon runs and was renowned as one of the major salmon-producing streams of the Sacramento Valley (Yoshiyama et al. 2010). From 1940 to 1959, annual runs of 10 000–86 000 fish were reported for fall and about 1000–4000 for spring Chinook salmon. Fall and spring Chinook spawned largely in different regions of the river which thus promoted isolation by distance in addition to isolation by time.

In recent decades, the majority of Chinook salmon production in the Feather River has been heavily supported by hatchery production. Since 2001, both spring- and fall-run Chinook salmon escapement to the Feather River Hatchery (FRH) has averaged approximately 16 000 fish, while river returns (natural spawners) averaged approximately 79 000 fish. As a result, approximately 82% of the natural fall and 91% of the natural spring Chinook spawners in the Feather River basin are considered to be hatchery-origin fish. Available data show that age 3- and 4-year-old fish comprise the majority of both the spring and fall runs in this system (Cavallo et al. 2009).

**Figure 1:**
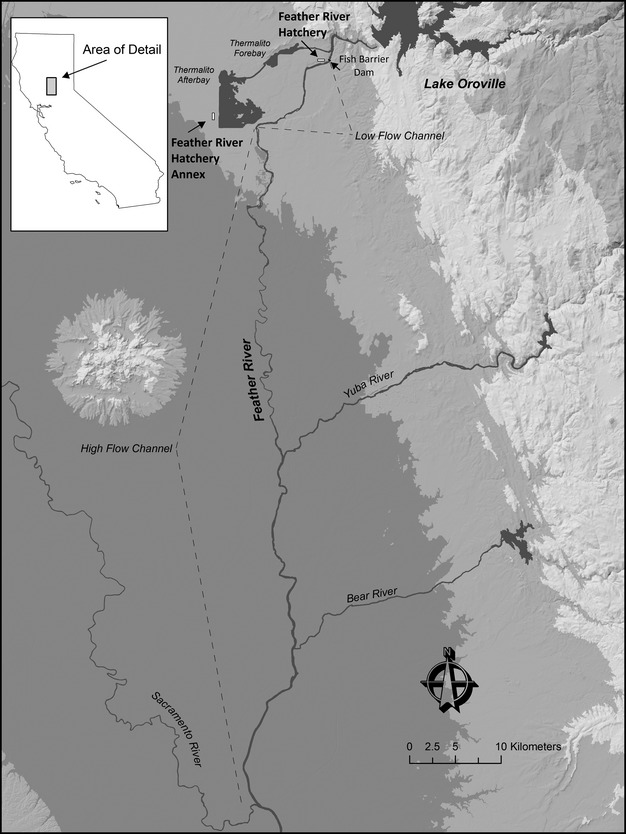
The Feather River Hatchery facility and area map showing its location in central California, USA.

For decades, separation of spring- and fall-run Chinook salmon at the FRH was based on arrival time such that fish arriving in September were spawned as spring run and those arriving in October were spawned as fall run. Beginning in 2006, a tagging program was implemented to ensure that only phenotypically spring-run fish were used in the spring-run broodstock. Subsequently, any fish that returns to the hatchery in May and June receives a pair of uniquely numbered external dart tags (Hallprint Fish Tagging Solutions) prior to release back into the river. Only hallprint-tagged Chinook salmon are collected in September and used as spring-run broodstock. Approximately, 750 females and 750 males are needed annually to meet the FRH spring-run Chinook salmon production target of releasing up to 2 million juveniles. Fall-run broodstock consists of nonhallprint-tagged fish that are ripe after October 1. The FRH is unable to hallprint tag every fish that arrives in May and June as not all fish enter the hatchery (Fig. 1). As a result, there is continued mixing of phenotypic spring-run fish into the fall-run broodstock. The degree to which this occurs can be monitored each year given that 100% of spring and 25% of fall-run hatchery-produced juveniles receive a coded wire tag (CWT) prior to release. To minimize crossing of run types, multiple lots from the fall run have been culled, which has dramatically reduced the number of spring-run CWT fish included in the fall-run production. Without 100% marking of the fall-run hatchery fish, wild, or hatchery origin among FRH broodstock cannot be accurately assessed.

In 2009, we combined CWT data (which assigns run type to juveniles at release) and hallprint tag data (which assigns run type to returning adults) to identify four adult migratory groups returning to spawn in this river system: spring return spring (spring) (CWT, hallprint tag), fall return fall (fall) (CWT), offspring of fall-run fish that returned in the spring (FRS) (CWT, hallprint tag), and offspring of spring-run fish that returned in the fall (SRF) (CWT) (Table 1). Considering that SRF are offspring of known spring-run parents (identified by 2006 hallprint tag), it is likely that a proportion of this migratory group consisted of early arriving nonhallprint-tagged fish in 2009. Given the uncertainty of individual adult run time phenotype, we excluded the SRF group from further analyses. Fin clips were obtained from the 2009 returning adult Chinook salmon: spring (*N* = 50; 22 males and 28 females), fall (*N* = 44; 21 males and 23 females), and FRS (*N* = 50; 25 males and 25 females).

**Table 1:** Four migratory groups of Chinook salmon that returned to the Feather River, CA, in 2009. Run identification was determined using both coded wire tag (CWT) and hallprint tag (HP) data

Four migratory groups	Spring	Fall	Fall return spring (FRS)	Spring return fall (SRF)
2006 Offspring release	Spring (CWT)	Fall (CWT)	Fall (CWT)	Spring (CWT)
2009 Adult return	Spring (HP)	Fall	Spring (HP)	Fall

### Genetic analyses

#### Microsatellite loci

We genotyped individuals (*N* = 144) at ten microsatellite loci that have previously been used to discriminate among migratory runs of Chinook salmon in other California Central Valley river systems (Banks et al. 2000). Primer sequences, GenBank accession numbers, and references are listed for each locus in Appendix A, Table A1. We extracted genomic DNA from fin clip tissue using the protocol derived by Ivanova et al. (2006) and performed polymerase chain reaction (PCR) amplification in 5 μL reactions according to the authors' protocols. Products were electrophoresed on an ABI 3730XL DNA Fragment Analyser and scored using GeneMapper® software (Applied Biosystems, Foster City, CA, USA).

#### Adaptive gene markers

We used six circadian clock gene markers and one spawning time QTL-linked marker to test for differentiation among the fall, spring, and FRS migratory groups: *OtsClock1b*, *Cryptochrome2b.2*, *Cryptochrome2b.3*, *Cryptochrome3*, *OmyFbxw11, Omy1009UW*, and *Ots515NWFSC*. Primer sequences, GenBank accession numbers, and references for the studies that first isolated each gene marker in salmon are listed in Appendix A, Table A2.

*OtsClock1b* is a single amino acid repeat-containing protein (SARP) (Siwach et al. 2006) with a polyglutamine repeat motif (PolyQ) localized to the C-terminal portion of the protein. *OtsClock1b* PolyQ length variation in Chinook salmon is primarily characterized by insertion and deletions consisting of both glutamine (Q) and proline (P) repeats (O'Malley and Banks 2008a). We used previously designed primers to amplify the *OtsClock1b* PolyQ domain in Feather River Chinook and test for frequency differences in length polymorphisms among migratory groups (O'Malley et al. 2007). In addition, we examined associations between *OtsClock1b* PolyQ mean allele length (MAL) and adult return time. MAL is defined as the sum of allele lengths of individuals in a population sample divided by the sample size (O'Malley and Banks 2008b).

We used published primers to amplify microsatellite repeat motifs in noncoding regions of the four additional circadian clock gene markers: *Cryptochrome2b.2*, *Cryptochrome2b.3*, *Cryptochrome3*, and *OmyFbxw11*. *Cryptochromes* are a family of blue light-sensitive flavoproteins that mediate circadian rhythms in plants and animals as well as affect sensing of magnetic fields in a number of species (Reppert and Weaver 2002). *OmyFbxw11* has been identified as a putative F-box protein in rainbow trout. Members of the F-box protein family have been shown to direct the ubiquitination and degradation of CRYPTOCHROME proteins in mammals (Dardente et al. 2008). *Omy1009UW* is a microsatellite locus linked to the circadian clock gene, *NPas2,* while the microsatellite *Ots515NWFSC* is linked to quantitative trait loci for spawning time and body weight in rainbow trout (Appendix A, Table A2). PCR products were electrophoresed on an ABI 3730XL DNA Fragment Analyser and scored as length polymorphisms using GeneMapper® software.

### Statistical analyses

Conformance to Hardy–Weinberg equilibrium (HWE) was examined using Genepop version 3.3 (Raymond and Rousset 1995). Number of observed alleles per locus and expected and observed heterozygosity were calculated using Genetix version 4.02 (Belkhir et al. 2000). Overall association among samples was assessed through factorial correspondence of analysis (FCA). Factorial correspondence of analysis is an exploratory technique, suitable for categorical data, which allows investigation of correspondence between rows (i.e., individuals) and columns (i.e., alleles) in a two-way table. It enables graphical visualization of individuals in multidimensional space, with no *a priori* assumptions about grouping, using each allele as an independent variable. Axes are generated from combinations of alleles that explain portions of the total observed ‘inertia’ of the table. Hence, those alleles exhibiting the strongest nonrandom association with groups of individuals will contribute most to the axes.

We performed an exact test for differences in genic frequencies among samples with specified Markov chain parameters of 5000 dememorization steps followed by 500 batches of 2000 iterations per batch (Genepop version 3.3). This exact test for population differentiation is accurate and unbiased even for very small samples or low-frequency alleles (Raymond and Rousset 1995). We calculated pairwise *F*_st_ values (Weir and Cockerham 1984) to estimate the level of genetic variation among the three migratory groups and used a permutation test with 1000 iterations to assess the statistical significance (Genetix Version 4.02).

## Results

### Neutral marker differentiation

Significant deviations from HWE were found at *OtsG78b* in all three population samples and at *Ots209* in the spring-run sample (Table 2a). As an excess of homozygotes was detected in all three population samples, we excluded *OtsG78b* from further analyses. We found no evidence for significant differentiation between the fall and spring migratory groups of Chinook salmon based on data from the nine presumably neutral microsatellite loci (Table 3a). Similarly, the pairwise *F*_st_ estimate and exact test for genic differentiation were not significant for the spring versus fall return spring (FRS) migratory group comparison. The fall versus FRS comparison, which served as a positive control, showed that the two migratory groups were genetically homogenous (Table 3a).

**Table 2:** Summary statistics for (a) ten microsatellite loci and (b) seven adaptive markers including number of individuals (*N*), number of alleles observed at each locus (*N*_a_), and observed and expected heterozygosity (*H*_o_ and *H*_e_) from each of the three migratory groups of Chinook salmon

(a)												
	Fall				Spring				FRS			
Locus	*N*	*N*_a_	*H*_o_	*H*_e_	*N*	*N*_a_	*H*_o_	*H*_e_	*N*	*N*_a_	*H*_o_	*H*_e_
*Ots104*	43	23	0.934	0.925	50	18	0.940	0.915	50	20	0.960	0.916
*Ots107*	43	20	1.000	0.930	50	21	0.940	0.920	50	21	0.940	0.921
*Ots211*	44	22	0.977	0.925	50	22	0.840	0.910	50	21	0.980	0.919
*OtsG409*	44	25	0.955	0.933	50	31	0.960	0.949	50	32	0.960	0.926
*Ots212*	44	24	0.977	0.946	50	22	0.900	0.906	50	22	1.000	0.929
*OtsG78b*	43	27	0.651	0.950	50	30	0.780	0.941	50	30	0.760	0.944
*Ots201b*	44	17	0.841	0.894	49	19	0.918	0.886	50	21	0.860	0.893
*Ots209*	44	25	0.977	0.933	49	25	0.939	0.943	50	25	0.980	0.937
*OtsG249*	44	29	0.955	0.936	50	27	0.960	0.949	50	30	0.960	0.949
*OtsG253b*	44	25	0.955	0.942	49	23	0.939	0.930	50	27	0.960	0.940
Average	43.7	23.3	0.954	0.930	49.7	23.1	0.926	0.923	50.0	24.3	0.956	0.926
(b)												
	Fall				Spring				FRS			
Gene marker	*N*	*N*_a_	*H*_o_	*H*_e_	*N*	*N*_a_	*H*_o_	*H*_e_	*N*	*N*_a_	*H*_o_	*H*_e_
*OtsClock1b*	40	3	0.475	0.462	49	3	0.531	0.452	50	3	0.500	0.479
*OmyFbxw11*	44	4	0.523	0.580	49	3	0.531	0.464	50	3	0.438	0.531
*Omy1009UW*	43	35	0.814	0.956	49	38	0.959	0.957	35	27	0.743	0.920
*Ots515NWFSC*	44	12	0.705	0.855	50	16	0.88	0.891	50	11	0.64	0.836
*Cryptochrome 2b.2*	44	5	0.818	0.719	45	6	0.778	0.705	50	5	0.800	0.703
*Crytpchrome 2b.3*	41	26	0.781	0.940	49	24	0.694	0.937	50	26	0.740	0.935
*Cryptochrome 3*	44	9	0.841	0.775	50	8	0.700	0.768	50	13	0.796	0.782

**Table 3:** Test statistics for measures of population differentiation among the three migratory groups based on variation at (a) nine microsatellite loci and (b) seven adaptive markers

(a)						
	Fall versus spring		Fall versus FRS		Spring versus FRS	
Locus	Genic	*F*_st_	Genic	*F*_st_	Genic	*F*_st_
*Ots104*	0.312	0.000	0.462	0.004	0.102	0.000
*Ots107*	0.090	0.000	0.345	0.000	0.003^*^	0.008^*^
*Ots211*	0.665	−0.001	0.710	−0.002	0.868	0.000
*OtsG409*	0.193	0.003	0.227	0.000	0.528	0.006^*^
*Ots212*	0.504	0.005	0.181	0.002	0.110	0.006
*Ots201b*	0.440	−0.003	0.513	−0.004	0.752	0.001
*Ots209*	0.259	0.001	0.307	0.004	0.345	−0.002
*OtsG249*	0.557	0.000	0.859	−0.001	0.907	−0.003
*OtsG253b*	0.155	0.001	0.595	−0.001	0.406	−0.001
*All*	0.235	0.002	0.608	0.000	0.088	0.002
(b)						
	Fall versus spring		Fall versus FRS		Spring versus FRS	
Gene marker	Genic	*F*_st_	Genic	*F*_st_	Genic	*F*_st_
*OtsClock1b*	0.012^*^	0.015	0.374	−0.003	0.229	−0.004
*OmyFbxw11*	0.018^*^	0.027^*^	0.123	−0.008	0.260	0.010
*Omy1009UW*	0.046^*^	0.002	0.321	0.001	0.238	0.002
*Ots515NWFSC*	0.256	−0.003	0.011^*^	0.000	0.012^*^	0.005
*Cryptochrome2b.2*	0.949	−0.008	0.991	−0.009	0.965	−0.008
*Cryptochrome2b.3*	0.104	−0.005	0.357	−0.006	0.109	−0.004
*Cryptochrome3*	0.431	−0.005	0.639	−0.002	0.023^*^	0.008
*All*	0.004^*^	0.001	0.123	−0.004	0.001^*^	0.001

Genic exact test *P*-values <0.05 are significant (^*^). *F*_st_ estimates with an associated *P*-value <0.05 are denoted significant (^*^).

### Adaptive marker differentiation

Based on variation at three circadian clock gene markers (*OtsClock1b*, *OmyFbxw11*, and *Omy1009UW*), we found evidence for significant differentiation between the spring and fall groups of Chinook salmon migrating to the Feather River in 2009. Results for the exact test at each marker were as follows: *OtsClock1b*, *P *=* *0.012; *OmyFbxw11*, *P *=* *0.018; *Omy1009UW*, *P *=* *0.046 (Table 3b). Only one marker pairwise *F*_st_ estimate was significant: *OmyFbxw11*, *F*_st_ = 0.027, *P *=* *0.042 (Table 3b). As predicted, spring and fall return spring (FRS) groups were not genetically distinct based on variation at the three clock gene markers. The spring versus FRS comparison served as a positive control since every 2009 returning adult from each group was identified as phenotypically spring-run migrant via the hallprint tag.

Contrary to prediction, the fall and FRS groups were genetically indistinguishable based on variation at *OtsClock1b*, *OmyFbxw11,* and *Omy1009UW* (Table 3b). Interestingly though, the MAL of the *OtsClock1b* PolyQ domain was the same for the both spring and FRS groups (345 base pairs) while the PolyQ MAL for the fall-run group was three base pairs shorter (342 base pairs); equivalent to one amino acid loss.

The spawning time and body weight QTL-linked marker, *Ots515NWFSC*, provided evidence for significant differentiation between the fall versus FRS as well as spring versus FRS migratory groups. Results for exact tests of genic differentiation were as follows: *F* versus FRS, *P *=* *0.011 and *S* versus FRS, *P *=* *0.012 (Table 3b). Pairwise *F*_st_ estimates were not significant. The three remaining circadian clock gene markers (*Cryptochrome2b.2*, *Cryptochrome2b.3,* and *Cryptochrome3*) did not discriminate between the fall and spring migratory groups (Table 3b).

**Figure 2:**
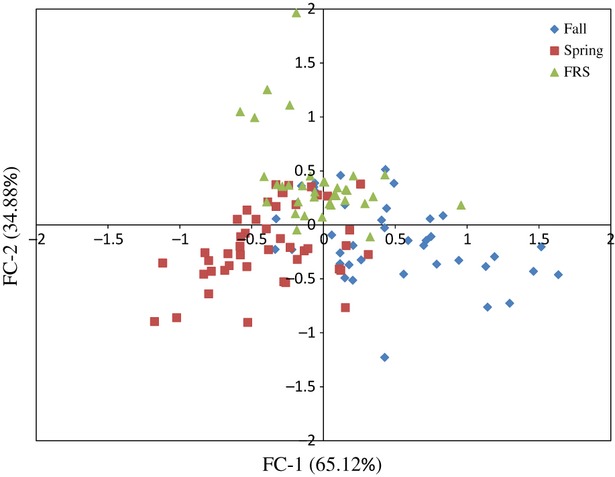
Factorial correspondence analysis based on data from three circadian clock gene markers (*OtsClock1b*, *OmyFbxw11,* and *Omy1009UW*) depicting the spatial representation of genetic differentiation among Chinook salmon migratory groups in the Feather River, California (fall ♦, spring ▪, and fall return spring ▴). Inertia of each axis is given in parentheses.

A factorial correspondence analysis based on data from the three diagnostic circadian clock gene markers (*OtsClock1b*, *OmyFbxw11* and *Omy1009UW*) shows separation between the fall and spring groups, predicted clustering of spring and FRS (positive control comparison) and unpredicted clustering of fall and FRS migrants (Fig. 2).

## Discussion

### Adaptive genetic markers discriminate migratory groups

Three circadian clock genes (*OtsClock1b*, *OmyFbxw11,* and *Omy1009UW*) differentiate between the fall and spring migratory groups of Chinook salmon amid gene flow high enough to homogenize unlinked neutral markers. Our results demonstrate the importance of marker type when attempting to delineate ESUs that have different adaptations and represent an important evolutionary legacy of a species. Furthermore, these findings suggest that the circadian clock genes may influence the migration timing of Feather River Chinook, which is consistent with previous studies of Pacific and Atlantic (*Salmo salar*) salmon (O'Malley et al. 2007, 2010a, 2013; O'Malley and Banks 2008b), and more generally, monarch butterflies (*Danaus plexippus*); the organism in which genetic components underlying migratory behavior were first identified (Froy et al. 2003).

Discernment of a third migratory group, fall return spring (FRS), provided an opportunity to further compare the discriminatory power of neutral versus adaptive genetic markers. Based on the microsatellite data, the FRS group was indistinguishable from the fall and spring groups. Contrary to our prediction, however, the three diagnostic circadian clock genes (*OtsClock1b*, *OmyFbxw11,* and *Omy1009UW)* failed to differentiate between the FRS and fall groups. A notable finding, however, showed that FRS and spring groups shared the same *OtsClock1b* PolyQ MAL, which was one amino acid longer than the fall-run MAL. This result is consistent with our previous study that reported longer *OtsClock1b* PolyQ MALs in early-migrating Chinook salmon populations distributed in the northern geographical range of this species compared to a shorter PolyQ MALs found in late-migrating populations in the southern range (O'Malley and Banks 2008b). Similar trends have been reported in Atlantic salmon (*Salmo salar*) (O'Malley et al. 2013) and cyprinids (Krabbenhoft 2012) with longer PolyQ alleles found at a higher frequency in earlier spawning individuals.

Migration is a complex phenomenon involving a suite of morphological, sensory, and physiologically adaptive traits that are inherited as components of a ‘migratory gene package’ (reviewed in Liedvogel et al. 2011). Given that the genetic architecture of the migratory package likely involves numerous genes, it may make it difficult to detect small, but significant, additive genetic variation effects on this quantitative trait (Liedvogel et al. 2009). This may be especially true for the FRS migratory group as there has been evidence for integration of phenotypically spring-run fish into the fall-run hatchery broodstock. Hybridization may have further impacted our ability to detect a genetic difference between the FRS and fall groups based on variation at the three diagnostic clock gene markers (*OtsClock1b*, *OmyFbxw11,* and *Omy1009UW*). The factorial correspondence analysis clearly illustrates the clustering of FRS and fall, while the fall and spring groups represent two distinct groups; likely attributed to the pure ancestry of the spring-run broodstock.

We did, however, find significant evidence for differentiation between the FRS and fall as well as the spring and FRS groups at *Ots515NWFSC*, a microsatellite linked to a spawning time and body weight QTL in rainbow trout (O'Malley et al. 2003). This marker may reflect variation at another adaptive trait that is part of the ‘migratory gene package' or it could represent a false positive and thus be biologically irrelevant. Given that this marker previously provided strong evidence for differentiation between migratory runs of Chinook salmon in two different systems (*F*_st_ = 0.058, *P *=* *0.003; *F*_st_ = 0.095, *P *=* *0; O'Malley et al. 2007), it warrants further investigation.

### Molecular mechanisms of the circadian clock

Several vertebrate systems (human, rodent, bird, frog, and fish) have been used in an effort to decipher the molecular mechanisms underlying the endogenous circadian clock pacemaker. The mouse has proven to be the most informative system demonstrating that the mammalian circadian clock is composed of an interconnected positive and negative transcriptional–translational feedback loop (reviewed in Takahashi et al. 2008). Six genes have been shown to function as key components of the mammalian central clock: *Clock* and its paralogue *Npas2*, *Bmal1*, *Period1,* and *Period2*, *Cryptochrome1* and *Cryptochrome2*. During the day, the transcription factor CLOCK (or NPAS2) interacts with BMAL1 to activate transcription of the *Period* and *Cryptochrome* genes, resulting in high levels of these transcripts. The resulting PERIOD and CRYPTOCHROMES proteins heterodimerize, translocate to the nucleus, and interact with the CLOCK–BMAL1 complex to inhibit their own transcription. During the night, the PERIOD–CRYPTOCHROME repressor complex is degraded, and CLOCK–BMAL1 can then activate a new cycle of transcription. The entire cycle takes approximately 24 h to complete.

Studies on the circadian clock system in teleosts have primarily been limited to zebrafish (Cermakian et al. 2000; Whitmore et al. 2000). Zebrafish possess homologues of both mammalian and invertebrate clock genes that exhibit comparable rhythmic expression patterns thus leading to the suggestion that the teleost clock systems may represent an evolutionary link (Pando and Sassone-Corsi 2002). Of the six circadian clock genes evaluated in this study, we found that the three involved in transcriptional activation (*OtsClock1b*, *OmyFbxw11*, and *Omy1009UW* linked to *Npas2*) proved to be diagnostic markers, while the three transcriptional repressors (*Cryptochrome2b.2*, *Cryptochrome2b.3,* and *Cryptochrome3*) did not discriminate between the fall and spring migratory groups. While the roles of *Clock* and *NPas2* in the interlocked feedback loops are well understood, studies have only recently shown that F-box proteins direct the degradation of CRYPTOCHROME and PERIOD proteins thereby ending repression of the CLOCK–BMAL1 complex (Busino et al. 2007; Siepka et al. 2007). As these studies have been limited to mammals, further research is required to determine the function of *OmyFbxw11* within the teleost circadian pacemaker.

Seasonal changes in day length have been shown to regulate expression patterns of the clock system in sheep (Lincoln et al. 2003), rat (Sumová et al. 2004), and Japanese quail (Yasuo et al. 2003). More recently, Davie et al. (2009) found that circadian clock gene expression in Atlantic salmon is day length dependent suggesting that the basic molecular mechanisms involved in the interpretation of seasonal day length changes might be conserved among vertebrates. Day length is known to strongly influence the physiology and behavior of salmon (Bromage et al. 2001). While previous studies have linked the circadian clock genes, *Clock, Bmal1,* and *Period1*, to temporal variations in spawning time in rainbow trout (Leder et al. 2006), migration timing in Chinook salmon (O'Malley et al. 2007; O'Malley and Banks 2008b), and reproductive strategy in Atlantic salmon (Aubin-Horth et al. 2005), the mechanisms by which photic information is perceived, interpreted, and then used to regulate many physiological seasonal events in salmon are unknown.

### Presumably neutral microsatellite locus

*OtsG78b* was one of ten microsatellite loci that showed significant deviation from HWE in all three population samples. Two factors that result in the observed excess of homozygotes include the presence of null alleles or selection. While our study does not attempt to differentiate between these two factors, it is interesting to note that *OtsG78b* has been shown to be associated with resistance to infectious hematopoietic necrosis virus (IHNV) in rainbow and steelhead trout (*O. mykiss*) (Rodriquez et al. 2004). IHNV, which causes severe necrosis of hematopoietic tissues including the anterior kidney, spleen, and pancreas, has been the primary disease concern at the FRH. While IHNV has continued to evolve in the FRH, the resulting strains do not appear to be more virulent than earlier ones. In concordance, epizootics causing IHNV had been a reoccurring problem up until 1998 (Cavallo et al. 2009). Deviation from HWE at *OtsG78b* and coupled with the recent decline in IHNV at the FRH suggests that this marker may be associated with disease resistance as has been reported for *O. mykiss*. Given these observations, further investigation is warranted to determine whether *OtsG78b* could be used as a diagnostic marker to study disease resistance in hatchery populations of salmon.

## Implications and conclusions

Here, we demonstrate the importance of genetic marker choice in resolving complex life history types involved in conservation management actions such as delineating evolutionary significant units. Conservation units described solely on the basis of divergence at neutral markers may exclude adaptively differentiated populations that warrant separate management. The three diagnostic clock markers identified here are components of an extensive molecular mechanism which thereby provides an opportunity to identify additional candidate genes for migration timing. Increasing the number of adaptive genetic markers used to resolve migratory groups in the Feather River may (i) help inform current hatchery practices and (ii) prove to be useful in a long-term monitoring program. First, employing a larger suite of adaptive markers may help facilitate identification of nonhallprint-tagged spring-run migrants that are inadvertently incorporated into the fall-run hatchery broodstock, which is currently not possible. Both genetic and CWT data could then be used to exclude early-returning migrants from the fall-run broodstock through real-time analyses. Second, neutral and adaptive markers could be incorporated into a long-term monitoring plan to estimate genetic divergence due to drift and adaptive differentiation among the migratory groups. This information could be used to assess the effectiveness of the FRH tagging program in preserving the phenotypic/genotypic characteristics of the threatened Feather River spring run. Ultimately, genomic data from both neutral and adaptive markers should be integrated to make optimal management decisions to conserve this spring run (Funk et al. 2012) that was recently selected as the primary source population for reintroduction to restore salmon populations in the mainstem of the San Joaquin River, California, a project estimated to cost 20 million dollars (Karrigan et al. 2010).

Broadly speaking, many conservation and habitat management strategies will benefit significantly from a basic understanding of the genetics of animal migration. Identifying the genetic components of migration timing will not only facilitate delineation of CUs, but it will also enable predictions as to how different migratory species might respond to climate variability and which may be especially vulnerable to a changing climate. This will permit estimates of the relative contributions of plastic and genetic response patterns of migratory species to climate change, which might differ both between and within species (Liedvogel et al. 2011).

## Figures and Tables

**Table A1:** List of the ten microsatellite markers used to genotype adult Chinook salmon samples. Primer sequences, accession number, and reference are provided for each locus

Locus	Primer sequence		Accession No.	Reference
*OtsG78b*	Forward	GTC CCT TGA ATT GAA TTG ATT AGA	AF393188	*
	Reverse	CAG CCT ACT GCA GTT CAA TAG ACT		
*Ots104*	Forward	GCA CTG TAT CCA CCA GTA	AF069676	†
	Reverse	GTA GGA GTT TCA TTT GAA TC		
*Ots107*	Forward	ACA GAC CAG ACC TCA ACA	AF069679	†
	Reverse	ATA GAG ACC TGA ATC GGT A		
*Ots201b*	Forward	CAG GGC GTG ACA ATT ATG C	NA	NA
	Reverse	TGG ACA TCT GTG CGT TGC		
*Ots209*	Forward	CCA AGT GAC CTG CTG TGT AGT TAC	AJ534367	‡
	Reverse	TCT CAG TTG GAC AGT GTA ACA GC		
*Ots211*	Forward	TAG GTT ACT GCT TCC GTC AAT G	AJ534361	‡
	Reverse	GAG AGG TGG TAG GAT TTG CAG		
*Ots212*	Forward	TCT TTC CCT GTT CTC GCT TC	AJ534362	‡
	Reverse	CCG ATG AAG AGC AGA AGA GAC		
*Ots249*	Forward	TTC TCA GAG GGT AAA ATC TCA GTA AG	AF393192	*
	Reverse	GTA CAA CCC CTC TCA CCT ACC C		
*Ots253b*	Forward	GAG CAG GCC GAG CAG GTG TCT	AF393193	*
	Reverse	AAT TGG GTC ATT AAG GCT CTG TGG		
*OtsG409*	Forward	GTA GCC ATT TGT GTC ACC ATC ATT	AF393196	*
	Reverse	CAT TCT CCT GCC TCA CAG AGT TTA		

* Williamson et al. (2002).

† Nelson and Beacham (1999).

‡ Greig et al. (2003).

**A2:** List of seven adaptive gene markers used to genotype adult Chinook salmon samples. Primer sequences, accession no., and reference are provided for each marker

Locus	Primer sequence		Accession No.	Reference
*OtsClock1b*	Forward	CCT GTG TTT GTC TCC AAC AGC A	DQ780894	*
	Reverse	CTG TCA CTG CGA AAT TAC AGT CCT		
*Cryptochrome 2b.2*	Forward	ACA TGC TCT GTG TTT CTC TCC	GU82614.1	†
	Reverse	AAC GCT CGC TCA CCA TAA AA		
*Cryptochrome 2b.3*	Forward	GGG AGA ATC AGA AGA GAT ACA T	GU82614.2	†
	Reverse	TAA CAT ACA GAC AGT AGT CAG ACA G		
*Cryptochrome 3*	Forward	CTG TAT TCT GTG CTG GTT GG	HQ658572	†
	Reverse	TGA GCC TCT ATG GAT CAA TAG		
*OmyFbxw11*	Forward	GCA GGG AGG GAG GAA TAA AG	EZ786349	NA
	Reverse	GCA ACA AGC CCA GTC TCT TC		
*Ots515NWFSC*	Forward	ACA GTG ATG GAG CTT GAT TC	AY042705	‡
	Reverse	ACG ATT TCT ATT TGT CTC CG		
*Omy1009UW*	Forward	GGA AAC AAG CCT GGA GAC AA	AY518332	§
	Reverse	GAC AAA GGC CTT CAG CAA AC		

* O'Malley et al. (2007).

† O'Malley et al. (2010b).

‡ Naish and Park (2002).

§ Spies et al. (2005).

## Data archiving statement

Raw data for this manuscript are available in Dryad, doi:10.5061/dryad.r28v2.
